# Factor-inhibiting hypoxia-inducible factor (FIH) catalyses the post-translational hydroxylation of histidinyl residues within ankyrin repeat domains

**DOI:** 10.1111/j.1742-4658.2011.08022.x

**Published:** 2011-02-23

**Authors:** Ming Yang, Rasheduzzaman Chowdhury, Wei Ge, Refaat B Hamed, Michael A McDonough, Timothy D W Claridge, Benedikt M Kessler, Matthew E Cockman, Peter J Ratcliffe, Christopher J Schofield

**Affiliations:** 1 Chemistry Research Laboratory and Oxford Centre for Integrative Systems Biology, University of OxfordUK; 2 Department of Pharmacognosy, Assiut UniversityEgypt; 3 Henry Wellcome Building for Molecular Physiology, University of OxfordUK

**Keywords:** 2-oxoglutarate-dependent dioxygenase, ankyrin repeat domain, factor inhibiting HIF, histidinyl hydroxylation, post-translational hydroxylation

## Abstract

Factor-inhibiting hypoxia-inducible factor (FIH) is an Fe(II)/2-oxoglutarate-dependent dioxygenase that acts as a negative regulator of the hypoxia-inducible factor (HIF) by catalysing β-hydroxylation of an asparaginyl residue in its C-terminal transcriptional activation domain (CAD). In addition to the hypoxia-inducible factor C-terminal transcriptional activation domain (HIF-CAD), FIH also catalyses asparaginyl hydroxylation of many ankyrin repeat domain-containing proteins, revealing a broad sequence selectivity. However, there are few reports on the selectivity of FIH for the hydroxylation of specific residues. Here, we report that histidinyl residues within the ankyrin repeat domain of tankyrase-2 can be hydroxylated by FIH. NMR and crystallographic analyses show that the histidinyl hydroxylation occurs at the β-position. The results further expand the scope of FIH-catalysed hydroxylations.

**Database:**

The coordinates for the structure have been deposited in the Protein Data Bank in Europe (PDBe; http://www.ebi.ac.uk/pdbe) under accession code 2y0i

**Structured digital abstract:**

- FIH and TNKS-1 hydroxylate by enzymatic study (View Interaction 1, 2)
- FIH and Tankyrase-2 bind by x-ray crystallography (View interaction)
- FIH and Tankyrase-2 hydroxylate by enzymatic study (View Interaction 1, 2, 3)
- FIH and TRPV4 hydroxylate by enzymatic study (View interaction)
- GABPB2 and FIH hydroxylate by enzymatic study (View interaction)

Factor-inhibiting hypoxia-inducible factor (FIH) is an asparaginyl hydroxylase that catalyses the β-hydroxylation of an Asn-residue in the C-terminal transcriptional activation domain of the hypoxia inducible factor and of highly conserved Asn-residues within the ubiquitous ankyrin repeat domain protein family. Here we report that FIH also catalyses the β-hydroxylation of histidinyl residues in the ankyrin repeat domain of tankyrase-2, further expanding the scope of FIH-catalysed hydroxylations.

## Introduction

Factor-inhibiting hypoxia-inducible factor (FIH) is an asparaginyl hydroxylase acting on hypoxia-inducible factor (HIF), a transcription factor that mediates the hypoxic response in humans. The FIH-catalysed hydroxylation of a conserved asparaginyl (Asn) residue within the C-terminal transcriptional activation domain (CAD) of HIF-α reduces the interaction of HIF with the transcriptional coactivator p300/cAMP-response-element-binding protein (p300/CBP) [1,2], thereby inhibiting HIF-mediated transcription. The requirement of molecular oxygen coupled to appropriate kinetic properties for catalysis by the FIH and HIF-prolyl hydroxylases is proposed to enable them to act as sensing components for the HIF system [3,4]. In addition to HIF-α, FIH also catalyses the hydroxylation of conserved Asn residues within the ubiquitous ankyrin repeat domain (ARD)-containing protein family [5–9]. ARDs are composed of a variable number of 33-residue repeats that individually fold into antiparallel α helices connected by a β hairpin/loop. The hydroxylated Asn residues are located within the loop that links individual ankyrin repeats. Asn hydroxylation of ARD protein stabilizes the stereotypical ARD fold [10,11]. Although the physiological function(s) of Asn hydroxylation of ARDs are unclear, proteomic and biochemical studies imply that intracellular ARD hydroxylation on Asn residues may be widespread [5,8]. Studies on FIH-catalysed ARD hydroxylation have defined a largely degenerate hydroxylation consensus with very few residues (−8, −1, +10 relative to the hydroxylation position) showing any substantial conservation, which is consistent with its ability to accommodate multiple ARD substrates [12,13]. However, to date, residues that are actually hydroxylated by FIH are limited to asparaginyl and in one case, ankyrinR, an aspartyl residue [14].

Tankyrase is a member of the poly-ADP-ribose polymerase family, which uses NAD^+^ as a cosubstrate to link ADP-ribose polymers to target proteins, resulting in a post-translational modification referred to as PARsylation [15]. Previous work has identified multiple FIH-dependent Asn hydroxylation sites in the tankyrase-2 ARD, which were observed to be hydroxylated to differing extents [8]. Here, we report the unexpected findings that two histidinyl residues in the tankyrase-2 ARD, located at analogous positions to the asparaginyl hydroxylation sites, are also substrates for FIH-catalysed β-hydroxylation. *In vitro* biochemical studies suggest that FIH may also catalyse His hydroxylation in other ARDs. The results expand the scope of potential 2-oxoglutarate (2OG) oxygenase-catalysed post-translational modifications.

## Results

### FIH hydroxylates His 238 and His 553 residues in tankyrase-2 ARD

**Figure 1:**
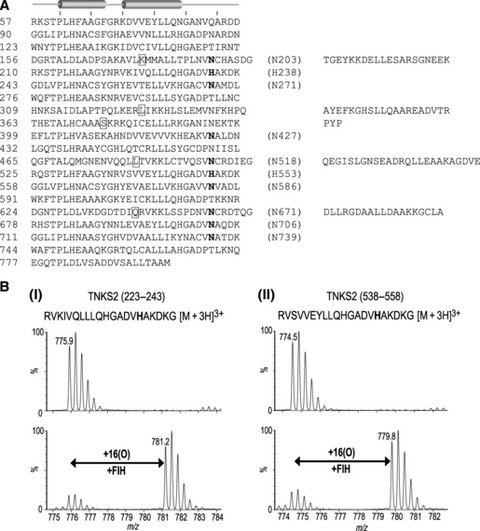
Tankryase-2 is a substrate for FIH-catalysed His hydroxylation. (A) Sequence alignment of the tankyrase-2 ARD (residues 57–798) [36] demonstrating a 4-repeat periodicity of the ARD, with insertion sequences (right) following boxed residues in the corresponding repeats to the left. Asn residues that have previously been identified as FIH substrates [8], and the two His residues located at the conserved hydroxylation position, are highlighted in bold and their residue number shown in parenthesis on the right. (B) Tankyrase-2 peptides containing His 238 and His 553 are FIH substrates *in vitro*. Peptides corresponding to: (I) TNKS2 223–243 (RVKIVQLLLQHGADVHAKDKG) and (II) TNKS2 538–558 (RVSVVEYLLQHGADVHAKDKG) were incubated in the presence of recombinant FIH and displayed a net +16 Da mass shift as determined by LC-MS. Subsequent MS/MS of the FIH-reacted TNKS2 538–558 peptide assigned the oxidation to His 553 (Fig. S1).

Previously, we have reported that various human ARD-containing proteins undergo hydroxylation at conserved Asn residues [5,6,8,9]. One of the most highly modified is tankyrase-2, which undergoes FIH-catalysed hydroxylation at eight Asn residues [8]. Alignment of the tankyrase-2 ARD revealed two His residues (His 238 and His 553) embedded within the FIH hydroxylation consensus comprising an ‘LxxxxxDVH’ motif at positions analogous to proven FIH-catalysed Asn hydroxylation sites (Fig. 1A) [8]. The positioning of these His residues within the hydroxylation consensus, coupled with the structural similarity between Asn and His residues, raised the interesting possibility that His 238 and/or His 553 of tankyrase-2 might also be hydroxylated by FIH. To test this hypothesis, we prepared synthetic 21-residue peptides encompassing the two His residues of interest and tested them as FIH substrates. Significantly, both peptides (TNKS2 223–243 and TNKS2 538–558) displayed a clear +16 Da mass increment after reaction with FIH under standard assay conditions (Fig. 1B). MS/MS analyses of the modified TNKS2 538–558 peptide after tryptic digestion assigned the site of hydroxylation to that corresponding to His 553 in the tankyrase-2 ARD (Fig. S1).

**Figure 2:**
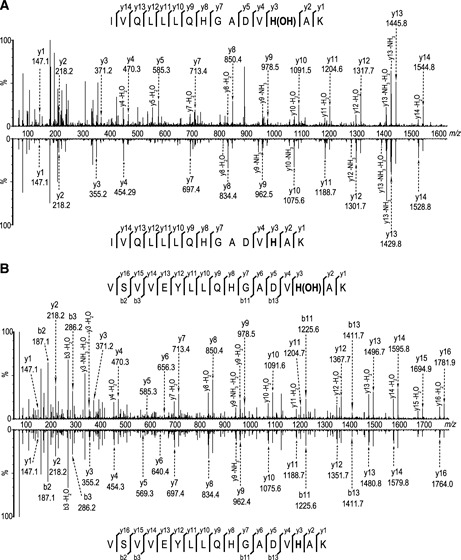
MS analyses assigning hydroxylation at His 238 and His 553 in tankyrase-2. Tankyrase-2 was purified from transiently transfected 293T cells coexpressing FIH. (A) MS/MS spectra of the tryptic peptide IVQLLLQHGADVHAK derived from tankyrase-2 (residues 226–240) in the hydroxylated ([M + 3H]^3+^ = *m/z* 553.28) (upper) and nonhydroxylated ([M + H]^3+^ = *m/z* 548.63) (lower) state. The hydroxylated species (upper) exhibits a + 16 Da mass shift on the y-ion series appearing at y3 and assigning hydroxylation to His238. (B) MS/MS of the tankyrase-2 tryptic peptide containing His 553 (VSVVEYLLQHGADVHAK) in hydroxylated ([M + 3H]^3+^ = *m/z* 627.64) (upper) and unmodified forms ([M + 3H]^3+^ = *m/z* 622.30) (lower). For both hydroxylated spectra, a −2 Da mass shift was commonly observed on fragment ions containing hydroxyhistidine, which is consistent with hydroxylation (+16 Da) followed by dehydration (−18 Da). Because there was no evidence for a −2 Da shift on the precursor ions it is likely that during the collision-induced dissociation process in the MS/MS analyses, the hydroxylated His residue undergoes dehydration to form the conjugated α,β-dehydrohistidine product. Note also there was no evidence for formation of the dehydrohistidine in the NMR analyses (Fig. 4).

Having established that His-containing peptides are FIH substrates *in vitro*, we then investigated whether tankyrase-2 might be subject to FIH-catalysed His hydroxylation in cells. To address this, we transfected plasmids encoding full-length FLAG-tagged tankyrase-2 and FIH into 293T cells, immunopurified the material by FLAG affinity and subjected it to trypsinolysis and MS/MS analysis. Peptides containing both His residues were observed, and MS/MS sequencing assigned hydroxylation at His 238 and His 553 (Fig. 2). As previously observed for hydroxyasparagine-containing peptides [8], under our HPLC conditions, the hydroxyhistidine modification had minimal effect on the peptide chromatographic properties and the hydroxylated and nonhydroxylated peptides coeluted (data not shown). The exact masses and retention times of the His-containing peptides were subsequently used to assign the hydroxylated and nonhydroxylated peptides studied by LC/MS analyses.

**Figure 3:**
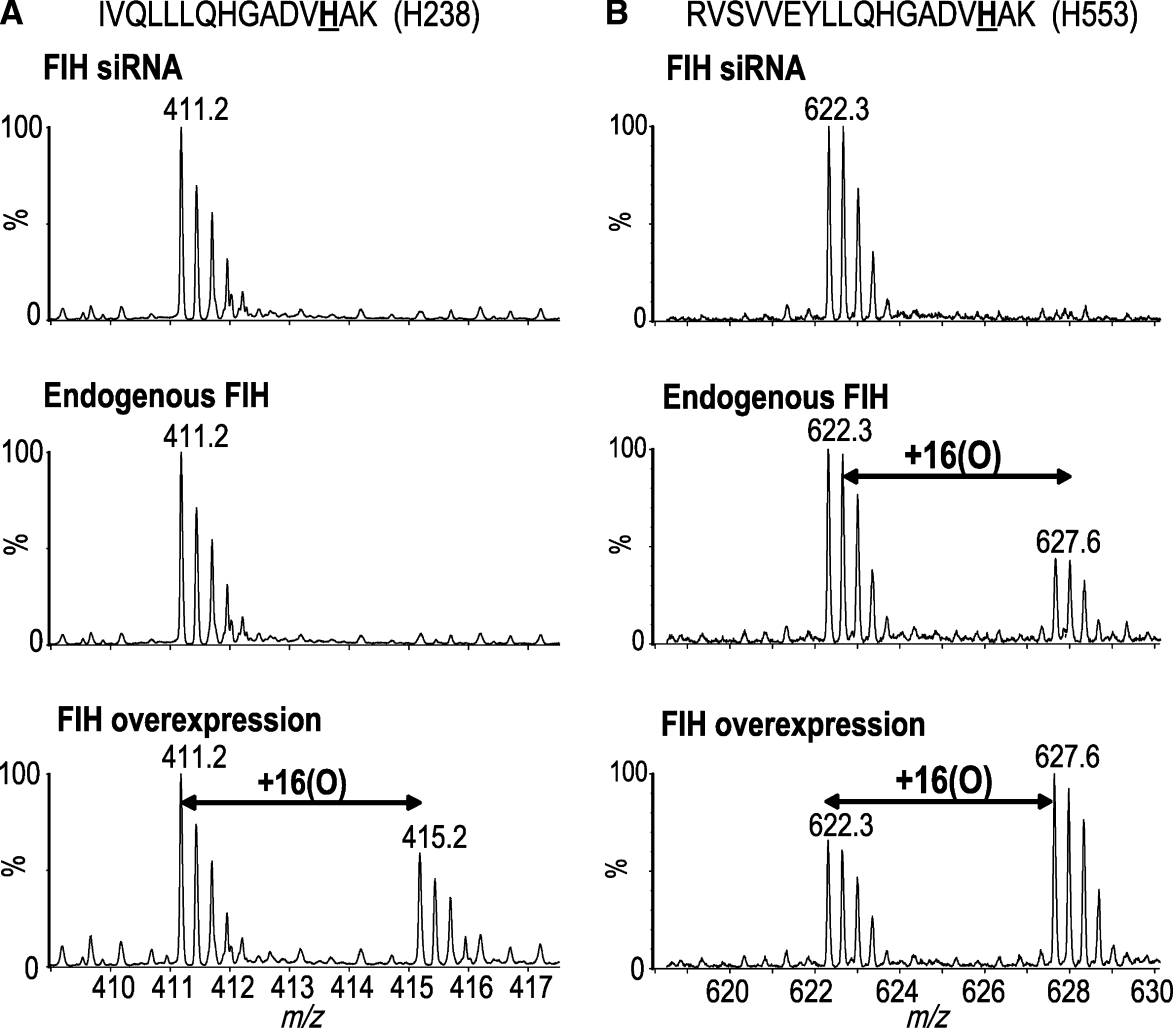
LC/MS spectra illustrating the effect of FIH intervention on tankyrase-2 hydroxylation at His 238 and His 553. 293T cells were treated with siRNAs against FIH (‘FIH siRNA’) or a control sequence (‘Endogenous FIH’/‘FIH overexpression’). After siRNA treatment, cells were transfected with FLAG–TNKS2 and either pcDNA3 FIH (‘FIH overexpression’) or empty vector (‘FIH siRNA’/‘Endogenous FIH’). FLAG–TNKS2 was immunopurified, digested and analysed by LC/MS to quantify hydroxylation. The efficacy of the siRNA and plasmid transfections were confirmed by anti-FIH and anti-FLAG immunoblotting (data not shown). (A) Representative LC/MS spectra of the 226–240 tryptic fragment containing His 238. Hydroxylation (∼ 30%) was observed for His 238 under conditions where the level of FIH was not limiting. (B) Representative LC/MS spectra of the 538–555 tryptic fragment containing His 553. Hydroxylation was observed at endogenous level of FIH (∼ 15%) and when FIH was overexpressed (∼ 70%).

To determine whether the His hydroxylation observed on tankyrase-2 was FIH dependent, we quantified hydroxylation at His 238 and His 553 by LC/MS in the presence and absence of small interfering RNA (siRNA) for FIH. 293T cells were transfected with siRNA duplexes directed against FIH or a nontargeting control, then transfected with tankyrase-2 plus empty vector. As an additional control, and to ensure that FIH levels were not limiting, tankyrase-2 was cotransfected with FIH. LC/MS data of one representative experiment are presented in Fig. 3. Following coexpression with FIH, the two hydroxylation sites displayed different levels of hydroxylation; His 238 was hydroxylated to ∼ 30%, whereas His 553 was hydroxylated to ∼ 70%. The preference for His 553 was also observed under physiological levels of FIH with detectable levels of hydroxylated peptide (∼ 15%) observed at His553, but no appreciable hydroxylation (< 4%) on the His 238 peptide. Importantly, siRNA-mediated knockdown of FIH suppressed His 553 hydroxylation to below the limit of detection, indicating a nonredundant role for FIH in the catalysis of hydroxyhistidine in the ARD of tankyrase-2. Consistent with previous work [8], the relative hydroxylation levels for some previously identified Asn hydroxylation sites in tankyrase-2 expressed in the presence of endogenous level of FIH were approximately: Asn 427, 12%; Asn 586, 42%; Asn 671, 5%; and Asn 739, 60% (tryptic fragments containing the Asn 203, Asn 271, Asn 518 and Asn 706 hydroxylation sites were not detected, data not shown).

### FIH-catalysed histidinyl hydroxylation occurs on the β-carbon

**Figure 4:**
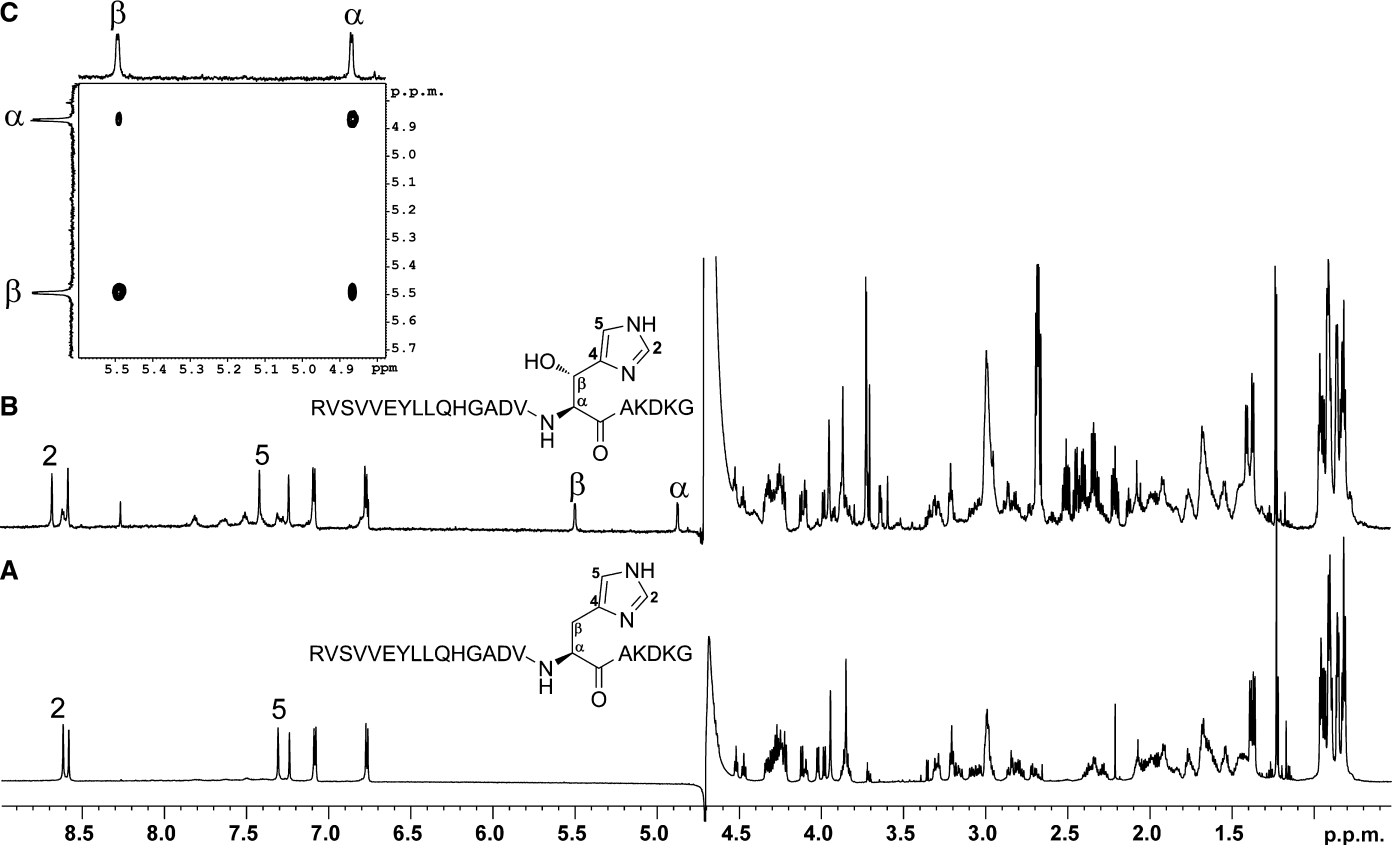
FIH catalysed His-hydroxylation occurs at the β-position. Hydroxylated TNKS2 538–558 peptide (RVSVVEYLLQHGADVHAKDKG) was produced by incubation with FIH under standard assay conditions (hydroxylated to ∼ 75% as assessed by MALDI-TOF analyses), LC-MS purified and analysed by NMR spectroscopy. (B) ^1^H NMR spectrum of the hydroxylated TNKS2 538–558 peptide in ^2^H_2_O. The resonances at 4.87 and 5.50 ppm, which are absent in the ^1^H NMR spectrum of the nonhydroxylated TNKS2 538–558 peptide (A), are ascribed to the α- and β-proton, respectively, of the hydroxylated His residues. The resonances for the imidazole ring protons (at positions 2 and 5) are deshielded in the spectrum of the hydroxylated peptide compared to that of the nonhydroxylated. (C) 2D ^1^H–^1^H COSY spectrum of the hydroxylated TNKS2 538–558 peptide in ^2^H_2_O indicating the ^1^H–^1^H correlation between resonances arising from the α- and β-hydrogens of the hydroxylated His residue.

FIH catalyses the hydroxylation of Asn residues at the β-position [16]. However, histidine residues are metal chelators and are prone to oxidation at their imidazole rings by reactive oxygen species, which are generated in a controlled manner during the catalytic cycle of 2OG-dependent dioxygenases [17]. Reactive oxygen species are also proposed to enable self-hydroxylation of FIH at an active-site tryptophan residue [18]. To investigate the regiochemistry of the FIH-catalysed His hydroxylation, we analysed the LC/MS-purified TNKS2 538–558 peptide product that had been hydroxylated by FIH (to ∼ 75%) using NMR spectroscopy. Compared with the spectrum of the nonhydroxylated peptide, two new doublet peaks at δ_H_ 4.87 and δ_H_ 5.50 ppm, which are coupled to each other (*J* = 3.0 Hz), were observed in the ^1^H spectrum of the hydroxylated TNKS2 538–558 peptide in ^2^H_2_O (Fig. 4). These resonances were assigned to the α- and β-protons, respectively, of the hydroxylated His 553 in the TNKS2 538–558 fragment. ^1^H–^13^C HSQC analysis (δ_H_α 4.87, δ_C_α 57.15 ppm) (δ_H_β 5.50, δ_C_β 65.15 ppm) supported this assignment (Fig. S2).

### Crystal structure of FIH bound to the tankyrase-2 fragment

**Figure 5:**
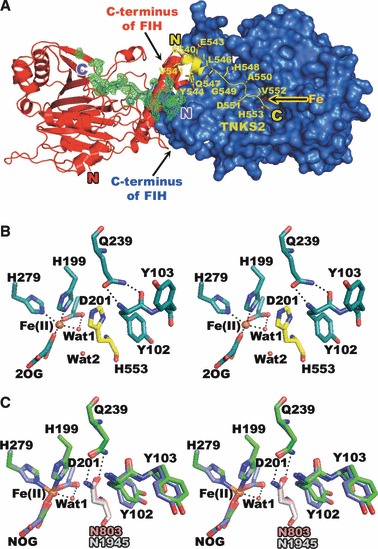
Structure of the FIH complexes. (A) Surface representation of the FIH·TNKS2 538–558 dimer structure (PDB ID: 2Y0I) to 2.28 Å resolution showing apparent electron density for residues Ser 540 to His 553 of the TNKS2 538–558 peptide (2*F*_o_ − *F*_c_ map, contoured to 1σ). (B) Stereoview stick representation of the FIH active site of the FIH·TNKS2 538–558 complex (FIH, deep teal; TNKS2 538–558, yellow; Fe(II), orange). (C) Stereoview stick representation of the superimposed FIH·mNotch1 1930–1949 (PDB ID: 3P3N, FIH in green and N1 1930–1949 in white) and FIH·HIF-1αCAD 788–826 (PDB ID: 1H2K; FIH in purple and HIF-1αCAD 788-826 in salmon) complexes. A comparison of all FIH complexes suggests that the pro-*S* hydrogen of His 553 in TNKS2 538–558 is likely analogously positioned as that observed for hydroxylated asparagines in FIH·mNotch1 1930–1949 (PDB ID: 3P3N) and FIH·HIF-1αCAD 788–826 (PDB ID: 1H2K) complexes. (B) and (C) also illustrate the differences in side-chain conformation for Gln 239_FIH_ and Tyr 103_FIH_ between the FIH·TNKS2 538–558 and FIH·mNotch1 1930–1949/FIH·HIF-1αCAD 788-826 complexes.

To investigate how a His residue is hydroxylated by FIH, we crystallized FIH in complex with Fe(II), 2OG and the TNKS2 538–558 fragment under anaerobic conditions [19]. The resultant overall FIH structure (2.28 Å resolution, Fig. 5A) was similar to reported FIH structures (rmsd values of 0.30–0.33 Å for Cα backbone atoms) and reveals that the backbone of TNKS2 538–558 is bound to FIH in a manner that is similar overall to analogous ARD fragments that undergo Asn hydroxylation and a fragment of the HIF-1α CAD substrate (rmsd for Cα backbone atoms of TNKS2 versus mNotch-1 and CAD ∼ 0.2 Å) [6,19]. At the N-terminus of the bound TNKS2 538–558 fragment, residues 541–546 form a short α-helix, possibly reflecting that in the ankyrin repeat (AR) of the parent tankyrase ARD protein (Fig. 5A). At the active site, the Fe(II) and 2OG are bound as first reported for the structure of FIH in complex with Fe(II) and a fragment of the HIF-1αCAD [20]. In the FIH·TNKS2 538–558 structure, the β-methylene of His 553 is positioned such that the pro-*S* hydrogen of its methylene group projects towards the Fe(II) centre (Fig. 5B), suggesting that it is hydroxylated to give the 3*S*-hydroxy product, as observed for Asn hydroxylation by FIH [16]. Histidine binding at the FIH active site apparently induces a stacking interaction between the substrate imidazole and the phenolic rings of Tyr 102_FIH_ and His 199_FIH_, which is one of the iron-complexing residues (Fig. 5B). Close to the β-methylene of His 553, we observed an electron density that was refined as a water molecule, although we cannot rule out the possibility that this density represents another species (e.g. partial reaction of the substrate; however, attempted refinements with hydroxylated His 553 were unsuccessful). The imidazole side chain of His 553 in the TNKS2 538–558 fragment points towards the γ-methylene of the side chain of Gln 239_FIH_ (Fig. 5B). Previous structures have shown that Gln 239_FIH_ binds via hydrogen-bonding interactions to the side chain of Asn residues that undergo hydroxylation, for example, Asn 803 of HIF-1α (Fig. 5C) [6,19]. However, in the TNKS2 538–558 structure, the side-chain amide of Gln 239_FIH_ is moved away from the side chain of the hydroxylated residue (i.e. with His 553 compared with a hydroxylated Asn residue) such that it is positioned to make a hydrogen bond with the backbone amide of Tyr 102_FIH_. Apparently concomitant with this change, the side chain of Tyr 103_FIH_ also moves (Fig. 5B).

### Evidence that FIH may catalyse His hydroxylation in other AR sequences

**Figure 6:**
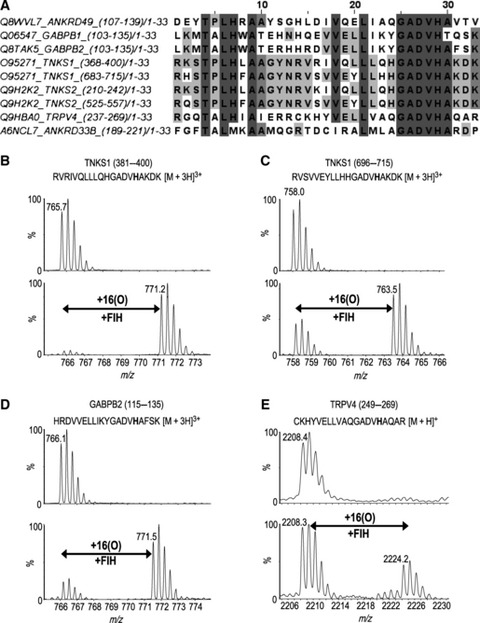
FIH-catalysed His hydroxylation of ARDs may be common. (A) clustalw nongapped multiple sequence alignment of ankyrin repeat sequences containing a target histidine residue at the conserved hydroxylation position. Corresponding peptides spanning the potential histidinyl hydroxylation sites were tested as FIH substrates *in vitro*, among which peptides derived from TNKS1, GABPB2 and TRPV4 demonstrate FIH-dependent hydroxylation. (B–D) LC/MS analyses demonstrating FIH-catalysed His hydroxylation of the following peptides: (B) TNKS1 381–400; (C) TNKS1 696–715; and (D) GABPB2 115–135. (E) MALDI spectra showing the hydroxylation of the TRPV4 249–269 peptide.

To investigate whether FIH-catalysed His hydroxylation can occur in other AR sequences, we searched for naturally occurring AR sequences containing an ‘LxxxxxDVH’ motif with the His residue located at the conserved hydroxylation position, and tested the corresponding peptides as FIH substrates (Fig. 6). In addition to tankyrase-2, peptides derived from tankyrase-1, GA-binding protein subunit beta-2 (GABPB2) and the transient receptor potential vanilloid-4 (TRPV4) ARD all displayed +16 Da mass shifts after reaction with FIH (Fig. 6B). MS/MS analyses assigned sites of hydroxylation to His 711 in the tankyrase-1 sequence (Fig. S3) and His 265 in the TRPV4 sequence (Fig. S4), both of which are located within the β-hairpin loop at the structurally conserved hydroxylation position.

To investigate the relative efficiency of FIH-catalysed histidinyl versus asparaginyl and aspartyl hydroxylations under standard conditions, we individually replaced the His residue at the hydroxylation position in the TNKS2 538–557 peptide with an Asn (TNKS2_H553N 538–557) or Asp residue (TNKS2_H553D 538–557) (Fig. S3). The three tankyrase-2-derived AR peptides were then incubated with FIH under identical assay conditions, under which the TNKS2_H553N 538–557 peptide was hydroxylated to near completion (> 95%), the TNKS2_H553D 538–557 peptide was hydroxylated to ∼ 90%, and the His-containing TNKS2 538–557 peptide was hydroxylated to ∼ 60% (Fig. S5). These observations support the proposals arising from analyses with intact tankyrase-2 protein that FIH-catalysed hydroxylation of His residues is less efficient compared with that of Asn or Asp residues, at least within the same sequence background.

## Discussion

The results, with both peptide fragments and intact tankyrase-2 protein in human cells, demonstrate that FIH can catalyse β-hydroxylation of His residues. Quantification of the extent of hydroxylation on the tankyrase-2 protein revealed that His hydroxylations are not as prevalent as some Asn hydroxylations, raising the possibility that His hydroxylation is less efficient. Consistent with this, peptide studies comparing the efficiency of FIH-catalysed hydroxylations on otherwise identical His-, Asp- and Asn-containing peptides (based on a tankyrase-2 peptide containing His 553) indicated a preference for Asn at the target position (Fig. S5). However, it is important to note that the efficiency of FIH-catalysed ARD hydroxylation depends not only on the primary sequence and the target residue, but also on the position of the AR, and on the overall fold [8,10,11]. Thus, it is possible that for other proteins FIH-catalysed His hydroxylation is more efficient.

We were able to demonstrate that endogenous FIH levels are sufficient to catalyse His hydroxylation of ectopically expressed tankyrase-2, but with the available antibodies we were unable to purify sufficient endogenous tankyrase-2 for analysis of the modification on the native protein. Histidine hydroxylation was reproducibly observed at one site (His 553) on transfected tankyrase-2 under physiological levels of FIH expression, demonstrating site-specific selectivity. When we have been able to purify and quantitate endogenous ARD substrates, such as with IκBα [5], Notch-1 [6] and MYPT-1 [9], and compare the levels of hydroxylation with their 293T transfected counterparts, the data from the two expression systems are in good agreement. Indeed, we have found that, in these cases, the level of hydroxylation in protein obtained from transfected cells tends to under-represent the true extent of the observed endogenous modification, possibly as a result of limiting FIH activity, supporting the proposed existence of His hydroxylation in cells.

2-OG-dependent dioxygenases catalyse a very wide range of oxidative reactions, possibly the widest of any enzyme family [21]. In animals, however, the known reactions that they catalyse are limited to hydroxylations and *N*-methyl demethylations via hydroxylation of methyl groups. In plants and micro-organisms, they catalyse a much wider range of reactions, including hydroxylation and desaturation (e.g. such as occurs in flavonoid biosynthesis) [22,23]. The observation that FIH can catalyse histidinyl β-hydroxylation is therefore of interest. Together with the recent findings that FIH can catalyse hydroxylation of an aspartyl residue in ankyrinR [14], the results presented here suggest that the substrate selectivity of FIH may be even wider than previously perceived. The combined results also raise the possibility that FIH homologues from the JmjC family of 2OG dioxygenases may have hydroxylation substrates other than asparaginyl, aspartyl or lysyl residues.

From a structural perspective, the observations that FIH has flexibility in the residues that it can oxidize is interesting because, in terms of sequence selectivity, FIH is known to be highly promiscuous. The crystallographic analyses suggested how FIH can accommodate both amide and imidazole side chains. The hydroxylated histidine is positioned such that its β-carbon can undergo hydroxylation, as observed for asparaginyl substrates of FIH. However, binding of the imidazole ring of the substrate histidine is different to that observed for the amide of asparaginyl substrates. The nitrogens of the histidinyl imidazole are not positioned to form hydrogen bonds (∼ 4 Å to O^ε^ Gln 239_FIH_). Instead the imidazole ring is sandwiched between the side chains of His 199_FIH_ that forms part of the catalytic triad and the aromatic ring of Tyr 102_FIH_. The side chain of Gln 239_FIH_ moves away from the substrate to form a hydrogen bond with the backbone of Tyr 102_FIH_, with concomitant movement of Tyr 103_FIH_ (Fig. 5). The difference in how FIH accommodates Asn and His residues at the active site likely accounts in part for the fact that His residues are less efficient substrates than Asn residues for hydroxylation. Mutagenesis studies on the importance of individual residues in the HIF-1αCAD in FIH catalysis have shown that only the hydroxylated Asn residue is essential [24], and combined studies on ARD substrates imply that FIH probably accepts many (perhaps > 100) human ARs as substrates. Further, FIH accepts both unstructured substrates, for example, the HIF-α or individual AR sequences, and structural ARD proteins as substrates. The promiscuity of FIH is further emphasized by the work described here.

The physiological significance of FIH-catalysed His hydroxylation is currently unclear. Taken together, it is conceivable that His hydroxylation might exert a physiological function either independent of, or in concert with, any of the eight previously assigned hydroxyasparagine modifications [8]. It may be that β-hydroxylation serves to stabilize the ARD fold [10,11] of tankyrase and, in doing so, modulates the hypoxic response along with other ARDs by regulating the amount of FIH that is bound to ARDs and therefore unavailable for hydroxylation of HIF-α [6,13]. It is also possible that Asn/His hydroxylation may modulate protein–protein interactions of tankyrase, as proposed for other FIH-catalysed hydroxylations [25]. An attractive possibility is that His hydroxylation plays a more direct functional role in redox signalling. Precedent for this comes from work on the ferric uptake repressor, PerR in *Bacillus subtilis*, which is inactivated by oxidation of the imidazole rings of two Fe(II)-binding histidinyl residues resulting in suppression of peroxide-defence genes [17,26]. β-Hydroxyhistidine is also present in both the bleomycin (Fig. S6) and nikkomycin antibiotics (Fig. S7), which are biosynthesized by nonribosomal peptide synthetases [27–29]. However, the stereochemistry of the β-hydroxyhistidine derivatives present in both bleomycin and nikkomycin is 2*S*,3*R*- rather than the likely 2*S*,3*S-*stereochemistry of β-hydroxyhistidine residues produced by FIH catalysis [16]. Further, in both of these antibiotics, biosynthesis is not catalysed by 2OG dioxygenases, revealing that nature has developed more than one route to this unusual amino acid residue.

## Materials and methods

### Peptide synthesis

Peptides were prepared using an Intavis Multipep automated peptide synthesizer with Tentagel-S-RAM resin (Rapp-Polymere, Tübingen, Germany) and a standard 9-fluorenylmethoxycarbonyl/*N*,*N*′-diisopropylcarbodiimide/1-hydroxybenzotriazole strategy. Final cleavage using 2.5% triisopropylsilane in CF_3_COOH yielded the peptides as C-terminal amides which were precipitated in cold ether, re-dissolved in 0.1% aqueous CF_3_COOH in water and then freeze-dried. The masses of the predicted peptide products were confirmed using a Micromass MALDI-TOF (Waters Manchester, UK) mass spectrometer.

### FIH purification and hydroxylation assays

Recombinant human FIH (with an N-terminal His6 tag) was produced in *Escherichia coli* BL21 (DE3) cells and purified by nickel-affinity chromatography and size-exclusion chromatography as reported previously [2]. *In vitro* FIH incubation assays employed 20–60 μm FIH, 100 μm Fe(II), 100 μm peptide, 1 mm 2OG and 1 mm ascorbate. The assay mixtures were incubated at 37 °C for 1 h prior to analysis.

### Cell culture and transfection

HEK293T cells were passaged in Dulbecco’s modified Eagle’s medium supplemented with 10% fetal calf serum (Sigma, St. Louis, MO, USA), 50 IU·mL^−1^ penicillin, 50 μg·mL^−1^ streptomycin and 2 mm l*-*glutamine. Prior to plasmid transfection, and where appropriate, the FIH gene was silenced by delivery of siRNA specific to human FIH (target F1) or nontargeting dHIF (*Drosophila* HIF) control. Cells were transfected twice at 24 h intervals using a 25 nm dose of duplex and Oligofectamine reagent (Invitrogen, Carlsbad, CA, USA) in accordance with the manufacturer’s instructions. Following siRNA delivery, cells were transfected with FuGENE6 (Roche, Welwyn Garden City, UK) in dishes of 15 cm diameter using 10 μg of total plasmid DNA in accordance with the manufacturer’s instructions. Cotransfection of tankyrase-2 (pFLAG/TNKS2) [8] with FIH (pCDNA3/FIH) [6] or empty vector control (pcDNA3) was performed at a ratio of 4 : 1 and cells were left for 48 h before downstream analysis.

### Immunoprecipitation and immunoblotting

Cells were lysed in IP buffer (20 mm Tris/HCl, pH 7.4, 100 mm NaCl, 5 mm MgCl_2_, 0.5% (v/v) Igepal CA-630) supplemented with 1 × Complete protease inhibitor cocktail (Roche) and subject to anti-FLAG immunoprecipitation using FLAG (M2) affinity gel (Sigma). FLAG-tagged tankyrase-2 was eluted in 0.5 m ammonium hydroxide (pH 10.5) and either resolved by SDS/PAGE and digested ‘in-gel’ or desalted by methanol/chloroform precipitation prior to ‘in-solution’ digestion with trypsin as described previously [8]. To confirm the efficacy of the siRNA-mediated knockdown and the plasmid co-transfection, input samples were subject to SDS/PAGE and immunoblotted with antibodies directed against FLAG-epitope (FLAG M2-HRP; Sigma) or FIH (clone 162c [30]).

### Mass spectrometry

Tryptic digest of tankyrase-2 purified from 293T cells were analysed by nanoUPLC-MS/MS using a 75 μm inner diameter, 25 cm length C_18_ nano-Acquity™ UPLC™ column (1.7 μm particle size; Waters) and a 90 min gradient of 2–45% solvent B (solvent A: 99.9% H_2_O, 0.1% HCOOH; solvent B: 99.9% MeCN, 0.1% HCOOH) on a Waters nanoAcquity UPLC system (final flow rate 250 nL·min^−1^; 6000–7000 psi) coupled to a Q-TOF Premier tandem mass spectrometer (Waters). MS analyses were performed in data-directed analysis mode (MS to MS/MS switching at precursor ion counts > 10 and MS/MS collision energy dependent on precursor ion mass and charge state). All raw MS data were processed with Proteinlynx Global Server software (plgs v. 2.2.5, Waters) including deisotoping. The mass accuracy of the raw data was calibrated using Glu-fibrinopeptide (200 fmol·μL^−1^; 700 nL·min^−1^ flow rate; 785.8426 Da [M + 2H]^2+^) that was infused into the mass spectrometer as a lock mass during sample analysis. MS and MS/MS data were calibrated at intervals of 30 s. Assignments of potential hydroxylation sites that were detected by plgs and mascot were evaluated and verified by manual inspection. For quantitative comparison of nonhydroxylated versus hydroxylated peptide peaks, the sum of all MS spectra containing the relevant precursor ion pairs are shown and the ratio was calculated by comparing the sum of ion counts for all isotopic peaks of the corresponding precursor ions. MS/MS analyses of synthetic peptides was performed on a Synapt™ high-definition MS (Micromass Ltd, Manchester, UK) using a 2.1 × 100 mm C_18_ Acquity UPLC® BEH300 column (1.7 μm particle size; Waters) and a 4 min gradient of 5–50% solvent B (solvent A: 99.9% H_2_O, 0.1% HCOOH; solvent B: 99.9% MeCN, 0.1% HCOOH) at a flow rate of 0.4 mL·min^−1^. LC/MS was performed at trap CE 6V and transfer CE 4V, and MS/MS at trap CE 35V and transfer CE 4V. MALDI-TOF MS analyses of synthetic peptides were performed on a Waters Micromass™ MALDI micro MX™ mass spectrometer in positive ion reflectron mode using α-cyano-4-hydroxycinnamic acid as the MALDI matrix. Instrument parameters used were: laser energy, 141%; pulse, 2050 V; detector, 2700 V; Suppression 1500.

### NMR analyses

The TNKS2538–558 peptide (RVSVVEYLLQHGADVHAKDKG) was hydroxylated (∼ 75%) by incubation with FIH in the presence of 2OG, Fe(II) and ascorbate at 37 °C for 4 h. The hydroxylation product was purified using a Vydac 218TP C_18_ reversed-phase column pre-equilibrated in 5% aqueous acetonitrile before running a gradient to 100% acetonitrile over 35 min. Elution was monitored using a Waters Micromass Quattro micro mass spectrometer (in positive ion mode) equipped with a Waters 2777 sample manager and a Waters 1525μ Binary HPLC pump system. Fractions with masses corresponding to anticipated product were collected (5–10 mL) and lyophilized. The peptide was relyophilized after suspending in 700 μL ^2^H_2_O. For NMR analysis, the sample was dissolved in 75 μL of ^2^H_2_O and transferred to a 2 mm NMR tube using a hand centrifuge. NMR experiments were performed at 310 K using a Bruker AVIII 700 spectrometer equipped with an inverse TCI cryoprobe optimized for ^1^H observation and running topspin 2 software. HSQC spectra were collected using adiabatic 180° CHIRP pulses and TOCSY experiments employed the DIPSI-2 isotropic mixing scheme with mixing times of 120 ms. Spectra are referenced to the residual water solvent signal at δ_H_ 4.72 ppm.

### Crystallography

Crystals of FIH·TNKS 538–558·Fe(II)·2OG were obtained under near anaerobic atmosphere (*P*O_2_<0.1 ppm) using 1.6 m ammonium sulphate, 6% (V/V) PEG400, 0.1 m Hepes/Na pH 7.5 [19]. A dataset for a FIH·Fe(II) 2OG·TNKS 538–558 crystal was collected at the Diamond beamline I04 with an ADSC Q315 3 × 3 CCD detector and was processed with automated data reduction software xia2 [31] and scala (ccp4 suite) [32]. Structure was solved by molecular replacement using phaser (search model PDB ID 1H2K) and was refined with CNS [33]. Iterative cycles of model building in coot [34] and slowcool-simulated annealing refinement using the maximum-likelihood function and bulk-solvent modelling in CNS proceeded until the decreasing *R*/*R*_free_ no longer converged. procheck [35] was used to monitor the geometric quality of the model between refinement cycles and identify poorly modelled areas needing attention. For data collection and refinement statistics see Table S1.

## Abbreviations

AR: ankyrin repeat
ARD: ankyrin repeat domain
CAD: C-terminal transactivation domain of HIF-α
FIH: factor inhibiting HIF
HIF: hypoxia inducible factor
2OG: 2-oxoglutarate
siRNA: small interfering RNA

## Supporting Information

The following supplementary material is available:

**Fig. S1.** His 553 in TNK2538–558(RVSVVEYLLQHGADVHAKDKG) is hydroxylated by FIH. **Fig. S2.** 1D ^1^H NMR and 2D^1^H–^13^C HSQC analyses ofhydroxylation of the TNKS2538–558 peptide. **Fig. S3.** His 711 in TNKS1696–715(RVSVVEYLLHHGADVHAKDK) is hydroxylated by FIH. **Fig. S4.** His 245 in TRPV4249–269(RCKHYVELLVAQGADVHAQAR) is hydroxylated by FIH. **Fig. S5.** FIH-catalysed His-hydroxylation is less efficient than that of Asn and Asp hydroxylations. **Fig. S6.** The presence ofβ-hydroxyhistidine in the glycopeptide antibioticbleomycin. **Fig. S7.** Biosynthesis ofβ-hydroxyhistidine found in nikkomycin antibiotics. **Table S1.** Refinement statistics for theFIH·TNKS 538-558·Fe(II)·2OG crystalstructure. Please note: As a service to our authors and readers, this journal provides supporting information supplied by the authors. Such materials are peer-reviewed and may be re-organized for online delivery, but are not copy-edited or typeset. Technical support issues arising from supporting information (other than missing files) should be addressed to the authors.

## References

[1] Lando D, Peet DJ, Gorman JJ, Whelan DA, Whitelaw ML, Bruick RK (2002). FIH-1 is an asparaginyl hydroxylase enzyme that regulates the transcriptional activity of hypoxia-inducible factor. Genes Dev.

[2] Hewitson KS, McNeill LA, Riordan MV, Tian YM, Bullock AN, Welford RW, Elkins JM, Oldham NJ, Bhattacharya S, Gleadle JM (2002). Hypoxia-inducible factor (HIF) asparagine hydroxylase is identical to factor inhibiting HIF (FIH) and is related to the cupin structural family. J Biol Chem.

[3] Flashman E, Hoffart LM, Hamed RB, Bollinger JM, Krebs C, Schofield CJ (2010). Evidence for the slow reaction of hypoxia-inducible factor prolyl hydroxylase 2 with oxygen. FEBS J.

[4] Koivunen P, Hirsila M, Gunzler V, Kivirikko KI, Myllyharju J (2004). Catalytic properties of the asparaginyl hydroxylase (FIH) in the oxygen sensing pathway are distinct from those of its prolyl 4-hydroxylases. J Biol Chem.

[5] Cockman ME, Lancaster DE, Stolze IP, Hewitson KS, McDonough MA, Coleman ML, Coles CH, Yu X, Hay RT, Ley SC (2006). Posttranslational hydroxylation of ankyrin repeats in IkappaB proteins by the hypoxia-inducible factor (HIF) asparaginyl hydroxylase, factor inhibiting HIF (FIH). Proc Natl Acad Sci USA.

[6] Coleman ML, McDonough MA, Hewitson KS, Coles C, Mecinovic J, Edelmann M, Cook KM, Cockman ME, Lancaster DE, Kessler BM (2007). Asparaginyl hydroxylation of the Notch ankyrin repeat domain by factor inhibiting hypoxia-inducible factor. J Biol Chem.

[7] Ferguson JE, Wu Y, Smith K, Charles P, Powers K, Wang H, Patterson C (2007). ASB4 is a hydroxylation substrate of FIH and promotes vascular differentiation via an oxygen-dependent mechanism. Mol Cell Biol.

[8] Cockman ME, Webb JD, Kramer HB, Kessler BM, Ratcliffe PJ (2009). Proteomics-based identification of novel factor inhibiting hypoxia-inducible factor (FIH) substrates indicates widespread asparaginyl hydroxylation of ankyrin repeat domain-containing proteins. Mol Cell Proteomics.

[9] Webb JD, Muranyi A, Pugh CW, Ratcliffe PJ, Coleman ML (2009). MYPT1, the targeting subunit of smooth-muscle myosin phosphatase, is a substrate for the asparaginyl hydroxylase factor inhibiting hypoxia-inducible factor (FIH). Biochem J.

[10] Kelly L, McDonough MA, Coleman ML, Ratcliffe PJ, Schofield CJ (2009). Asparagine beta-hydroxylation stabilizes the ankyrin repeat domain fold. Mol Biosyst.

[11] Hardy AP, Prokes I, Kelly L, Campbell ID, Schofield CJ (2009). Asparaginyl beta-hydroxylation of proteins containing ankyrin repeat domains influences their stability and function. J Mol Biol.

[12] Cockman ME, Webb JD, Ratcliffe PJ (2009). FIH-dependent asparaginyl hydroxylation of ankyrin repeat domain-containing proteins. Ann NY Acad Sci.

[13] Schmierer B, Novak B, Schofield CJ (2010). Hypoxia-dependent sequestration of an oxygen sensor by a widespread structural motif can shape the hypoxic response – a predictive kinetic model. BMC Syst Biol.

[14] Yang M, Ge W, Chowdhury R, Claridge TDW, Kramer HB, Schmierer B, McDonough MA, Gong L, Kessler BM, Ratcliffe PJ (2010). Asparaginyl and aspartyl hydroxylation of the cytoskeletal ankyrin family is catalysed by factor inhibiting hypoxia-inducible factor (FIH). J Biol Chem.

[15] Hsiao SJ, Smith S (2008). Tankyrase function at telomeres, spindle poles, and beyond. Biochimie.

[16] McNeill LA, Hewitson KS, Claridge TD, Seibel JF, Horsfall LE, Schofield CJ (2002). Hypoxia-inducible factor asparaginyl hydroxylase (FIH-1) catalyses hydroxylation at the beta-carbon of asparagine-803. Biochem J.

[17] Traore DA, El Ghazouani A, Jacquamet L, Borel F, Ferrer JL, Lascoux D, Ravanat JL, Jaquinod M, Blondin G, Caux-Thang C (2009). Structural and functional characterization of 2-oxo-histidine in oxidized PerR protein. Nat Chem Biol.

[18] Chen YH, Comeaux LM, Eyles SJ, Knapp MJ (2008). Auto-hydroxylation of FIH-1: an Fe(II), alpha-ketoglutarate-dependent human hypoxia sensor. Chem Commun (Camb).

[19] Elkins JM, Hewitson KS, McNeill LA, Seibel JF, Schlemminger I, Pugh CW, Ratcliffe PJ, Schofield CJ (2003). Structure of factor-inhibiting hypoxia-inducible factor (HIF) reveals mechanism of oxidative modification of HIF-1a. J Biol Chem.

[20] Elkins JM, Ryle MJ, Clifton IJ, Dunning Hotopp JC, Lloyd JS, Burzlaff NI, Baldwin JE, Hausinger RP, Roach PL (2002). X-Ray crystal structure of *Escherichia coli* taurine/alpha-ketoglutarate dioxygenase complexed to ferrous iron and substrates. Biochemistry.

[21] Flashman E, Schofield CJ (2007). The most versatile of all reactive intermediates?. Nat Chem Biol.

[22] Turnbull JJ, Prescott AG, Schofield CJ, Wilmouth RC (2001). Purification, crystallization and preliminary X-ray diffraction of anthocyanidin synthase from *Arabidopsis thaliana*. Acta Crystallogr D.

[23] Martens S, Preuss A, Matern U (2010). Multifunctional flavonoid dioxygenases: flavonol and anthocyanin biosynthesis in *Arabidopsis thaliana* L. Phytochemistry.

[24] Wilkins SE, Hyvarinen J, Chicher J, Gorman JJ, Peet DJ, Bilton RL, Koivunen P (2009). Differences in hydroxylation and binding of Notch and HIF-1alpha demonstrate substrate selectivity for factor inhibiting HIF-1 (FIH-1). Int J Biochem Cell Biol.

[25] Zheng X, Linke S, Dias JM, Gradin K, Wallis TP, Hamilton BR, Gustafsson M, Ruas JL, Wilkins S, Bilton RL (2008). Interaction with factor inhibiting HIF-1 defines an additional mode of cross-coupling between the Notch and hypoxia signaling pathways. Proc Natl Acad Sci USA.

[26] Lee JW, Helmann JD (2006). The PerR transcription factor senses H_2_O_2_ by metal-catalysed histidine oxidation. Nature.

[27] Du L, Sanchez C, Chen M, Edwards DJ, Shen B (2000). The biosynthetic gene cluster for the antitumor drug bleomycin from *Streptomyces verticillus* ATCC15003 supporting functional interactions between nonribosomal peptide synthetases and a polyketide synthase. Chem Biol.

[28] Calcutt MJ, Schmidt FJ (1994). Gene organization in the bleomycin-resistance region of the producer organism *Streptomyces verticillus*. Gene.

[29] Chen H, Hubbard BK, O’Connor SE, Walsh CT (2002). Formation of beta-hydroxy histidine in the biosynthesis of nikkomycin antibiotics. Chem Biol.

[30] Stolze IP, Tian YM, Appelhoff RJ, Turley H, Wykoff CC, Gleadle JM, Ratcliffe PJ (2004). Genetic analysis of the role of the asparaginyl hydroxylase factor inhibiting hypoxia-inducible factor (HIF) in regulating HIF transcriptional target genes. J Biol Chem.

[31] Winter G (2010). xia2: an expert system for macromolecular crystallography data reduction. J Appl Crystallogr.

[32] Bailey S (1994). The CCP4 suite: programs for protein crystallography. Acta Crystallogr D.

[33] Brunger AT, Adams PD, Clore GM, DeLano WL, Gros P, Grosse-Kunstleve RW, Jiang JS, Kuszewski J, Nilges M, Pannu NS (1998). Crystallography & NMR system: a new software suite for macromolecular structure determination. Acta Crystallogr D.

[34] Emsley P, Cowtan K (2004). Coot: model-building tools for molecular graphics. Acta Crystallogr D.

[35] Laskowski RA, MacArthur MW, Moss DS, Thornton JM (1993). PROCHECK: a program to check the stereochemical quality of protein structures. J Appl Crystallogr.

[36] Sbodio JI, Lodish HF, Chi NW (2002). Tankyrase-2 oligomerizes with tankyrase-1 and binds to both TRF1 (telomere-repeat-binding factor 1) and IRAP (insulin-responsive aminopeptidase). Biochem J.

