# Supplementary material for: Factor-inhibiting hypoxia-inducible factor (FIH) catalyses the post-translational hydroxylation of histidinyl residues within ankyrin repeat domains
Source: FEBS J. 2011 Feb 23;278(7):1086–97. doi: 10.1111/j.1742-4658.2011.08022.x (PMC3569879; doi:10.1111/j.1742-4658.2011.08022.x)
Supplement: Supplementary file 1 — Fig. S1. His 553 in TNK2538–558(RVSVVEYLLQHGADVHAKDKG) is hydroxylated by FIH. Fig. S2. 1D 1H NMR and 2D1H–13C HSQC analyses ofhydroxylation of the TNKS2538–558 peptide. Fig. S3. His 711 in TNKS1696–715(RVSVVEYLLHHGADVHAKDK) is hydroxylated by FIH. Fig. S4. His 245 in TRPV4249–269(RCKHYVELLVAQGADVHAQAR) is hydroxylated by FIH. Fig. S5. FIH-catalysed His-hydroxylation is less efficient than that of Asn and Asp hydroxylations. Fig. S6. The presence ofβ-hydroxyhistidine in the glycopeptide antibioticbleomycin. Fig. S7. Biosynthesis ofβ-hydroxyhistidine found in nikkomycin antibiotics. Table S1. Refinement statistics for theFIH·TNKS 538-558·Fe(II)·2OG crystalstructure. [file febs0278-1086-sd1.zip › febs_8022_sm_TableS1_FigsS1-S7.pdf]

the FEBS Journal
www.febsjournal.org

Factor-inhibiting hypoxia-inducible factor (FIH) catalyses the post-translational hydroxylation of histidinyl residues within ankyrin repeat domains

Ming Yang, Rasheduzzaman Chowdhury, Wei Ge, Refaat B. Hamed, Michael A. McDonough, Timothy D. W. Claridge, Benedikt M. Kessler, Matthew E. Cockman, Peter J. Ratcliffe and Christopher J. Schofield

DOI: 10.1111/j.1742-4658.2010.08022.x

Factor-Inhibiting Hypoxia-Inducible Factor (FIH) Catalyses the Posttranslational Hydroxylation of Histidinyl Residues within Ankyrin Repeat Domains

Ming Yang*, Rasheduzzaman Chowdhury*, Wei Ge*, Refaat B. Hamed*‡, Michael A. McDonough*, Timothy D. W. Claridge*, Benedikt M. Kessler†, Matthew E. Cockman†[1, 2], Peter J. Ratcliffe†[1] and Christopher J. Schofield*[1]

From *Chemistry Research Laboratory and Oxford Centre for Integrative Systems Biology, University of Oxford, Oxford OX1 3TA, UK, †Henry Wellcome Building for Molecular Physiology, University of Oxford, Oxford OX3 7BN, UK and ‡Department of Pharmacognosy, Faculty of Pharmacy, Assiut University, 71256, Egypt

[1] These authors contributed equally to this work.

[2] Address for correspondence: Matthew E. Cockman, Henry Wellcome Building for Molecular Physiology, University of Oxford, Oxford OX3 7BN, United Kingdom. Phone: +44 1865 287785; Fax: +44 1865 287787; Email: matthew@well.ox.ac.uk

Supplementary data

Fig. S1 His-553 in the $TNK2_{538-558}$ (RVSVVEYLLQHGADVHAKDKG) peptide is hydroxylated by FIH. (A) MS/MS of the unmodified $TNK2_{539-555}$ tryptic fragment. (B) MS/MS of the hydroxylated $TNK2_{539-555}$ tryptic fragment. A +16 Da mass shift (corresponding to hydroxylation) and a -2 Da shift (corresponding to hydroxylation followed by dehydration to form α, β-dehydrohistidine) was observed on y ions, starting at y_3 and thereby assigning hydroxylation at His-553 in $TNK2_{538-558}$. y^o denotes the corresponding y ion with a loss of water, likely during the MS/MS analysis.

Fig. S2 1D ^{1}H NMR and 2D 1H-^{13}C HSQC analyses of hydroxylation of the $TNKS2_{538-558}$ peptide. (A) ^{1}H NMR spectra showing the differences in chemical shifts of Hα of Val-552 and Hβ of Ala-554 between the hydroxylated and non-hydroxylated $TNKS2_{538-558}$ peptide. Upon hydroxylation, δHα of Val-552 moved from 4.02 ppm to 4.11 ppm and δHβ of Ala-554 moved from 1.38 ppm to 1.42 ppm. (B) 2D 1H-^{13}C HSQC analyses of the hydroxylated $TNKS2_{538-558}$ peptide. Resonances arising from the α- and β-hydrogens of the hydroxylated His-553 are indicated. No evidence was apparent for formation of a dehydrohistidinyl residue in the ^{1}H NMR spectrum.

Fig. S3 His-711 in the $TNKS1_{696-715}$ (RVSVVEYLLHHGADVHAKDK) peptide is hydroxylated by FIH. (A) MS/MS of the unmodified $TNKS1_{697-713}$ tryptic fragment. (B) MS/MS of the hydroxylated $TNKS1_{697-713}$ tryptic fragment. A +16 Da (corresponding to hydroxylation) as well as a -2 Da (corresponding to hydroxylation followed by dehydration) mass shift was observed on y ion series containing y_3, assigning hydroxylation to His-711 in $TNKS1_{697-713}$. y^o denotes the corresponding y ion with a loss of water likely during the MS/MS analysis.

Fig. S4 His-245 in the $TRPV4_{249-269}$ (RCKHYVELLVAQGADVHAQAR) peptide is hydroxylated by FIH. (A) MS/MS of the unmodified $TRPV4_{252-269}$ tryptic fragment. (B) MS/MS of the hydroxylated $TRPV4_{252-269}$ tryptic fragment. A +16 Da (corresponding to hydroxylation) as well as a -2 Da (corresponding to hydroxylation followed by dehydration) mass shift was observed on y ion series containing y_5, assigning hydroxylation at His-245 in $TRPV4_{249-269}$. y^o denotes the corresponding y ion with a loss of water.

Fig. S5 Comparing the reaction efficiency of the FIH-catalysed Asn-, Asp- and His-hydroxylations *in vitro* reveals that His hydroxylation is less favoured. The target His-residue in the $TNKS2_{538-557}$ peptide RVSVVEYLLQHGADVHAKDK (C) was replaced with either an Asn-residue ($TNKS2\_H553N_{538-557}$, RVSVVEYLLQHGADVNAKDK) (A) or an Asp-residue ($TNKS2\_H553D_{538-557}$, RVSVVEYLLQHGADVDAKDK) (B). The three peptides were tested as FIH substrates under

identical experimental conditions in the absence or presence of FIH. Data are representative of two independent experiments. (A) The Asn-substituted peptide TNKS2_H553N$_{538-557}$ is hydroxylated to near completion under standard assay conditions. (B) The Asp-substituted TNKS2_H553D$_{538-557}$peptide was hydroxylated to ~88%. (C) The wt His-containing peptide TNKS2$_{538-557}$ was hydroxylated to ~58%.

Fig. S6 The presence of β-hydroxyhistidine in the glycopeptide antibiotic bleomycin [1]. R = $NH(CH_2)_3S^+(CH_3)_2$, beomycin-A_2; R = $NH(CH_2)_4NHC(NH)NH_2$, bleomycin-B_2. The β-hydroxylation of the His-residue is considered to be catalysed by BlmORF1 [2], which takes place after the complete assembly of the polyketide backbone but before its release from the bleomycin megasynthetase [3].

Fig. S7 Biosynthesis of β-hydroxyhistidine found in Nikkomycin antibiotics. The β-hydroxylation reaction in Nikkomycin biosynthesis is catalysed by the heme-dependent monooxygenase NikQ. Figure adapted from Chen et al. [4]

References

1. Oppenheimer NJ, Rodriguez LO & Hecht SM (1979) Proton nuclear magnetic resonance study of the structure of bleomycin and the zinc-bleomycin complex. *Biochemistry* **18**, 3439-3445.
2. Sugiyama M, Thompson CJ, Kumagai T, Suzuki K, Deblaere R, Villarroel R & Davies J (1994) Characterisation by molecular cloning of two genes from Streptomyces verticillus encoding resistance to bleomycin. *Gene* **151**, 11-16.
3. Du L, Sanchez C, Chen M, Edwards DJ & Shen B (2000) The biosynthetic gene cluster for the antitumor drug bleomycin from Streptomyces verticillus ATCC15003 supporting functional interactions between nonribosomal peptide synthetases and a polyketide synthase. *Chem Biol* **7**, 623-642.
4. Chen H, Hubbard BK, O'Connor SE & Walsh CT (2002) Formation of beta-hydroxy histidine in the biosynthesis of nikkomycin antibiotics. *Chem Biol* **9**, 103-112.

Table S1

**Measurement**	**FIHQ239H.AnkR(Asp)**
**<u>Data Collection</u>**	
Space Group	$P4_12_12$
Cell dimensions a,b,c (Å)	86.49
	86.49
	146.71
Resolution (Å)	55.94-2.28 (2.34-2.28)*
No. of unique reflections	25989
Completeness (%)	99.6% (98.3%)*
Redundancy	9.3 (9.3)*
R_{sym}**	0.053 (0.774)*
Mean I/ σ(I)	23.9 (3.1)*
**<u>Refinement</u>**	
R_{factor}	0.2188
R_{free}	0.2433
R.m.s. deviation	
Bond length, Å	0.007
Bond angle, °	1.318
No. of atoms	
Protein	2775
Peptide	110
Ligand (2OG)	10
Water	114
$<B_{factor}>$Å^2	
Protein	55.88
Peptide	69.65
Ligand (2OG)	39.57
Metal (Fe)	44.68
Water	49.53

*Highest resolution shell shown in parenthesis

**$R_{sym} = \sum|I-<I>|/\sum I$, where I is the intensity of an individual measurement and $<I>$ is the average intensity from multiple observations.

Fig. S1

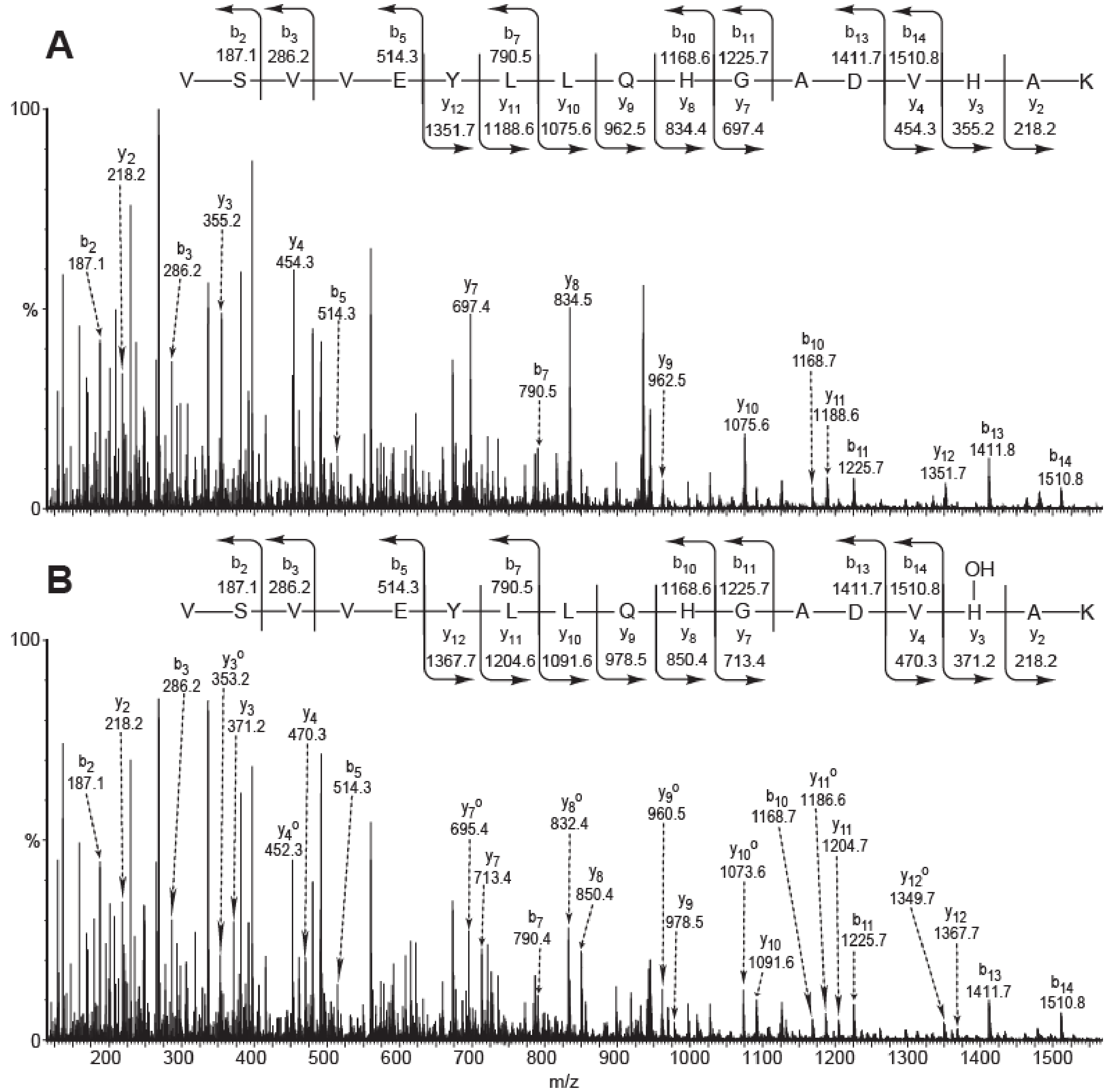
A
V—S—V—V—E—Y—L—L—Q—H—G—A—D—V—H—A—K
b2 187.1
b3 286.2
b5 514.3
b7 790.5
b10 1168.6
b11 1225.7
b13 1411.7
b14 1510.8
y12 1351.7
y11 1188.6
y10 1075.6
y9 962.5
y8 834.4
y7 697.4
y4 454.3
y3 355.2
y2 218.2
100
%
0
y2 218.2
b2 187.1
y3 355.2
b3 286.2
y4 454.3
b5 514.3
y7 697.4
y8 834.5
b7 790.5
y9 962.5
y10 1075.6
b10 1168.7
y11 1188.6
b11 1225.7
y12 1351.7
b13 1411.8
b14 1510.8
B
OH
V—S—V—V—E—Y—L—L—Q—H—G—A—D—V—H—A—K
b2 187.1
b3 286.2
b5 514.3
b7 790.5
b10 1168.6
b11 1225.7
b13 1411.7
b14 1510.8
y12 1367.7
y11 1204.6
y10 1091.6
y9 978.5
y8 850.4
y7 713.4
y4 470.3
y3 371.2
y2 218.2
100
%
0
b3 286.2
y3o 353.2
y2 218.2
y3 371.2
y4 470.3
b2 187.1
b5 514.3
y4o 452.3
y7o 695.4
y7 713.4
y8o 832.4
y8 850.4
b7 790.4
y9o 960.5
y9 978.5
b10 1168.7
y11o 1186.6
y11 1204.7
y10o 1073.6
y10 1091.6
y12o 1349.7
y12 1367.7
b11 1225.7
b13 1411.7
b14 1510.8
200 300 400 500 600 700 800 900 1000 1100 1200 1300 1400 1500
m/z

Fig. S2

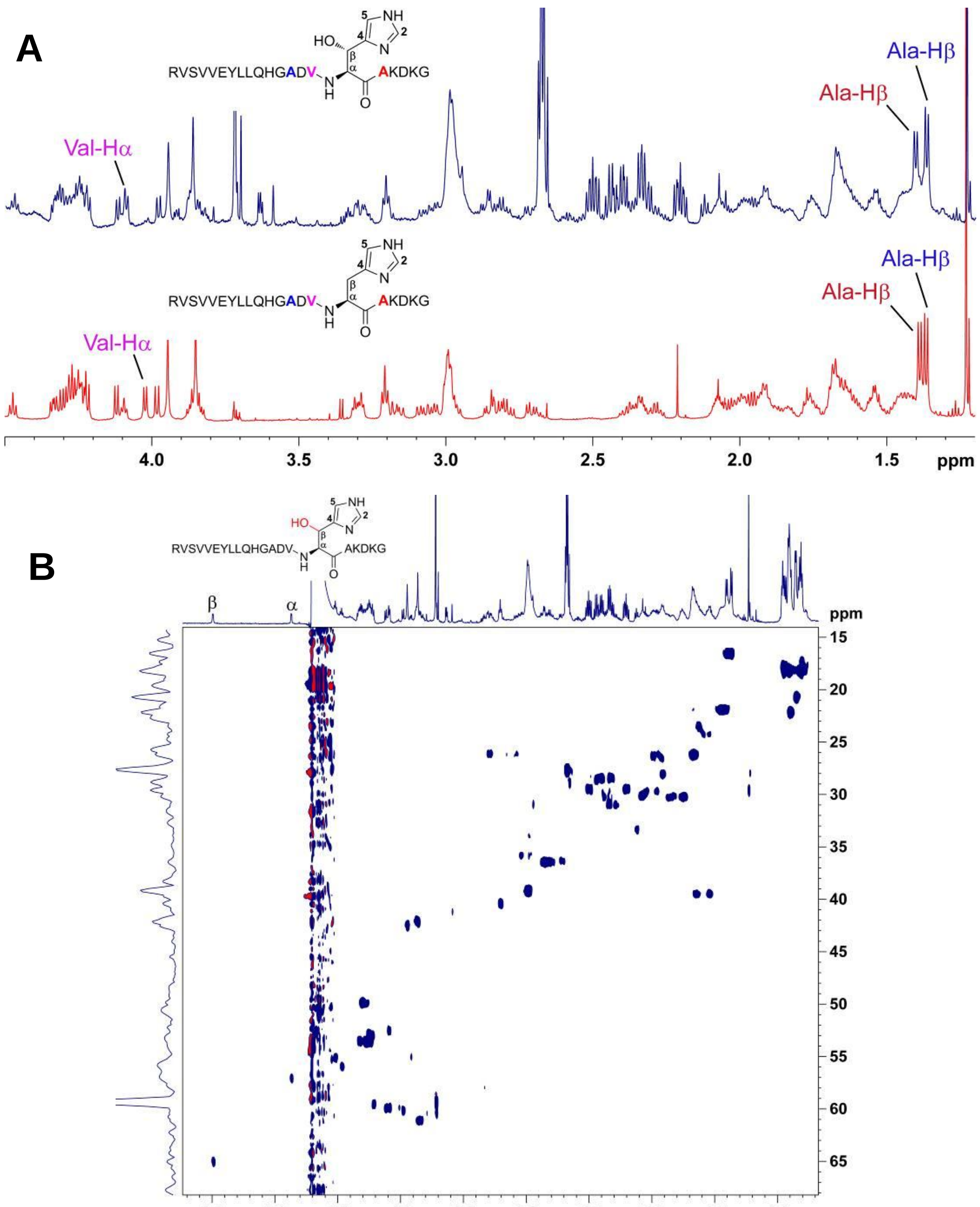
A
RVSVVEYLLQHGADV
AKDKG
HO
NH
N
H
O
5
4
2
β
α
Val-Hα
Ala-Hβ
Ala-Hβ
RVSVVEYLLQHGADV
AKDKG
Val-Hα
Ala-Hβ
Ala-Hβ
4.0
3.5
3.0
2.5
2.0
1.5
ppm
B
RVSVVEYLLQHGADV
AKDKG
HO
β
α
ppm
15
20
25
30
35
40
45
50
55
60
65
5.5
5.0
4.5
4.0
3.5
3.0
2.5
2.0
1.5
1.0
ppm

Fig. S3

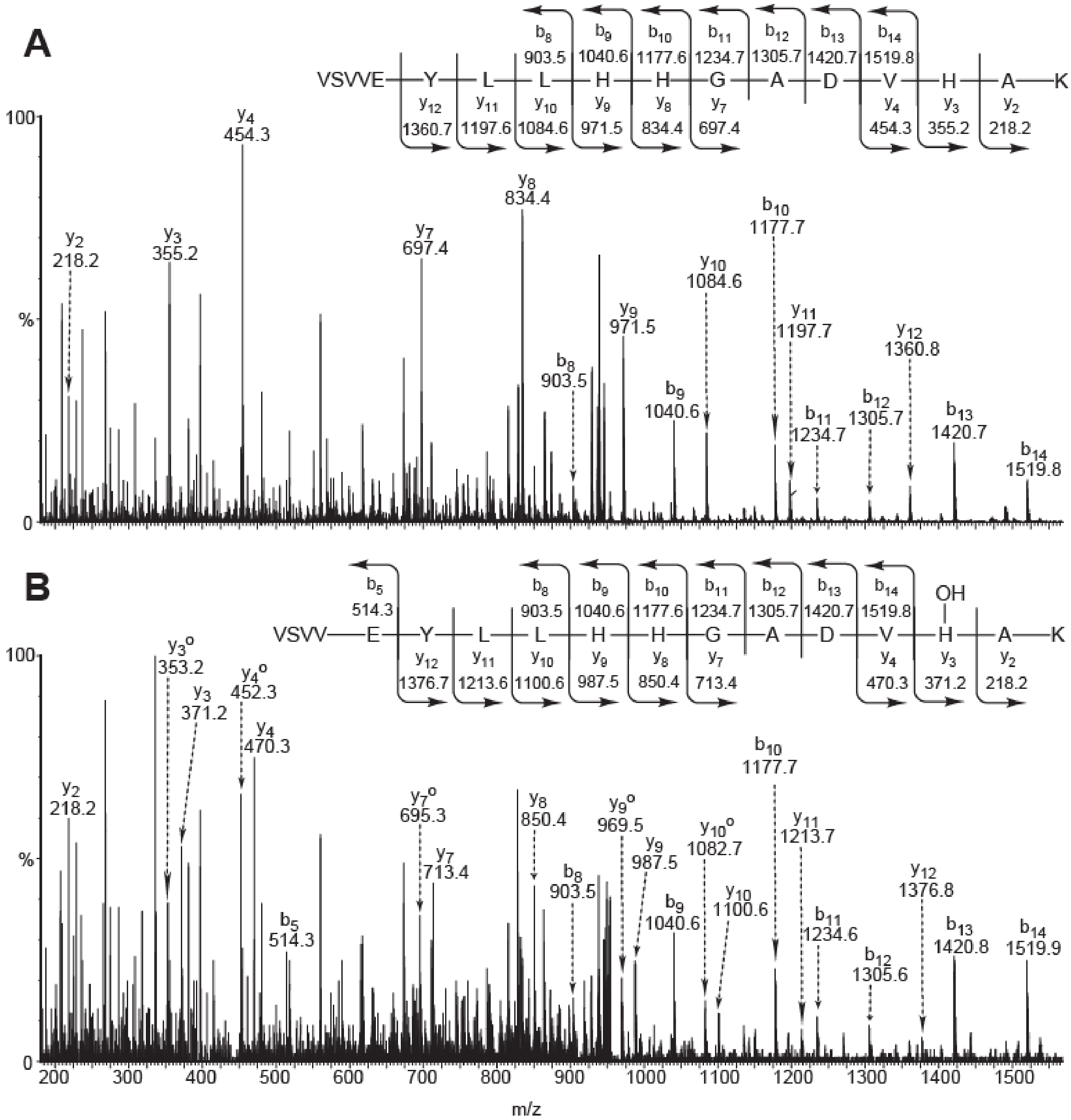
A
VSVVE—Y—L—L—H—H—G—A—D—V—H—A—K
b8 903.5
b9 1040.6
b10 1177.6
b11 1234.7
b12 1305.7
b13 1420.7
b14 1519.8
y12 1360.7
y11 1197.6
y10 1084.6
y9 971.5
y8 834.4
y7 697.4
y4 454.3
y3 355.2
y2 218.2
y2 218.2
y3 355.2
y4 454.3
y7 697.4
y8 834.4
b8 903.5
y9 971.5
b9 1040.6
y10 1084.6
b10 1177.7
y11 1197.7
b11 1234.7
b12 1305.7
y12 1360.8
b13 1420.7
b14 1519.8
B
VSVV—E—Y—L—L—H—H—G—A—D—V—H—A—K
OH
b5 514.3
b8 903.5
b9 1040.6
b10 1177.6
b11 1234.7
b12 1305.7
b13 1420.7
b14 1519.8
y12 1376.7
y11 1213.6
y10 1100.6
y9 987.5
y8 850.4
y7 713.4
y4 470.3
y3 371.2
y2 218.2
y2 218.2
y3o 353.2
y3 371.2
y4o 452.3
y4 470.3
b5 514.3
y7o 695.3
y7 713.4
y8 850.4
b8 903.5
y9o 969.5
y9 987.5
b9 1040.6
y10o 1082.7
y10 1100.6
b10 1177.7
y11 1213.7
b11 1234.6
b12 1305.6
y12 1376.8
b13 1420.8
b14 1519.9
100
%
0
200
300
400
500
600
700
800
900
1000
1100
1200
1300
1400
1500
m/z

Fig. S4

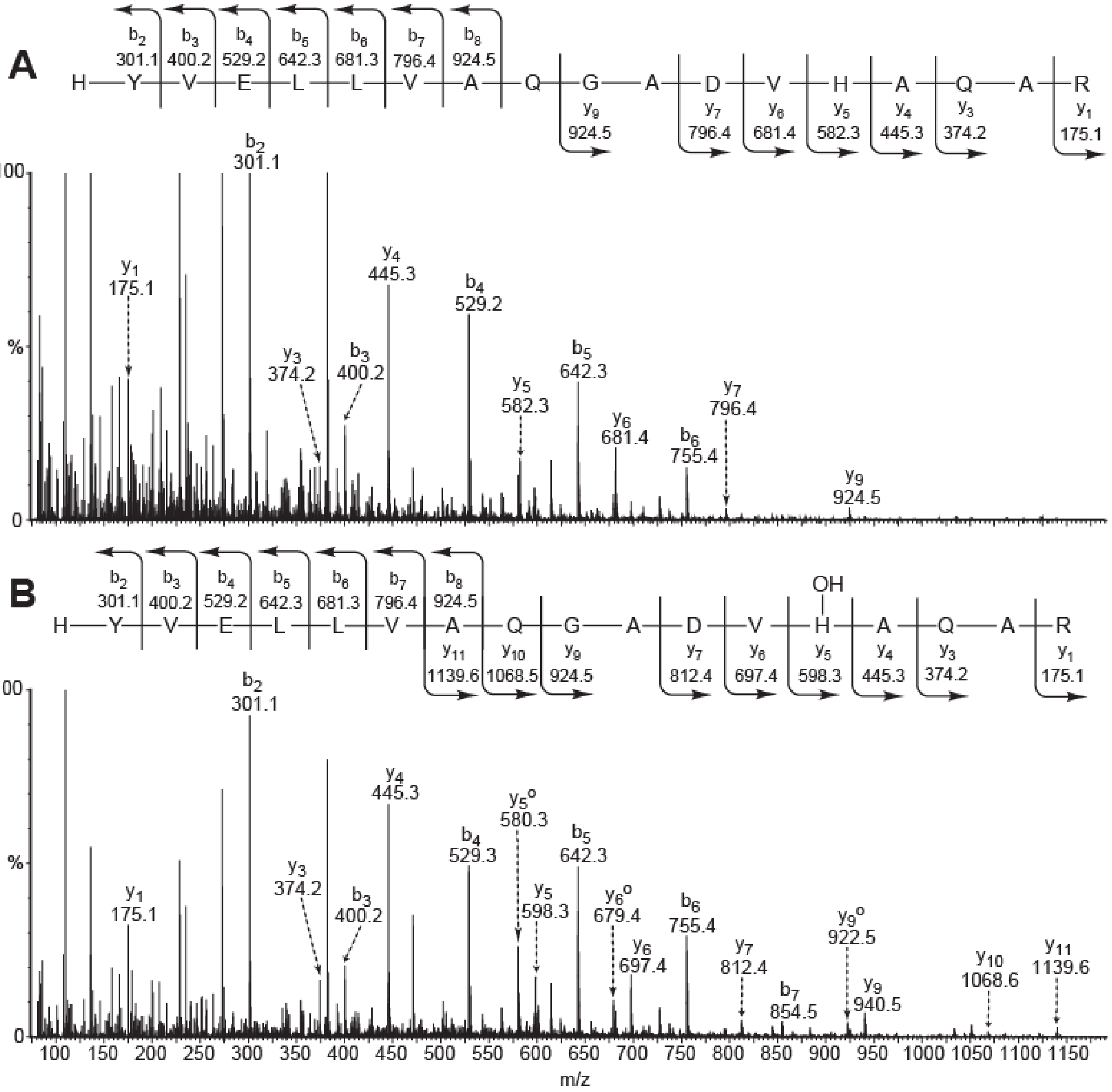
A
H—Y—V—E—L—L—V—A—Q—G—A—D—V—H—A—Q—A—R
b2 301.1
b3 400.2
b4 529.2
b5 642.3
b6 681.3
b7 796.4
b8 924.5
y9 924.5
y7 796.4
y6 681.4
y5 582.3
y4 445.3
y3 374.2
y1 175.1
100
%
0
b2 301.1
y1 175.1
y4 445.3
b4 529.2
y3 374.2
b3 400.2
b5 642.3
y5 582.3
y7 796.4
y6 681.4
b6 755.4
y9 924.5
B
OH
H—Y—V—E—L—L—V—A—Q—G—A—D—V—H—A—Q—A—R
b2 301.1
b3 400.2
b4 529.2
b5 642.3
b6 681.3
b7 796.4
b8 924.5
y11 1139.6
y10 1068.5
y9 924.5
y7 812.4
y6 697.4
y5 598.3
y4 445.3
y3 374.2
y1 175.1
100
%
0
b2 301.1
y4 445.3
y5o 580.3
b4 529.3
b5 642.3
y3 374.2
y1 175.1
b3 400.2
y5 598.3
y6o 679.4
b6 755.4
y6 697.4
y7 812.4
y9o 922.5
b7 854.5
y9 940.5
y10 1068.6
y11 1139.6
100 150 200 250 300 350 400 450 500 550 600 650 700 750 800 850 900 950 1000 1050 1100 1150
m/z

Fig. S5

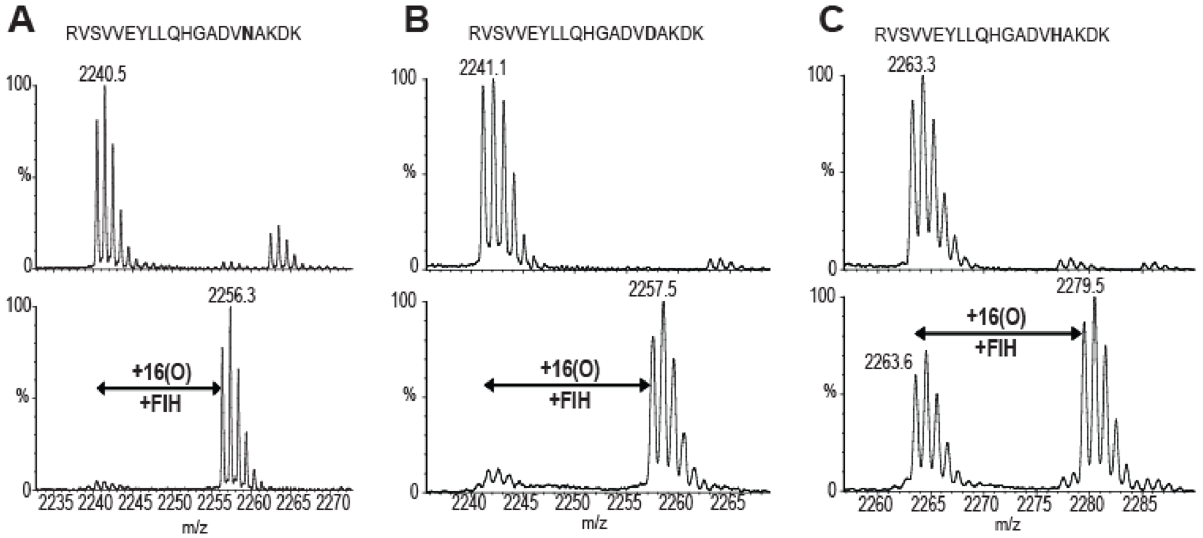

A
RVSVVEYLLQHGADVNAKDK
2240.5
100
%
0
2256.3
+16(O)
+FIH
2235 2240 2245 2250 2255 2260 2265 2270
m/z
B
RVSVVEYLLQHGADVDAKDK
2241.1
2257.5
+16(O)
+FIH
2240 2245 2250 2255 2260 2265
m/z
C
RVSVVEYLLQHGADVHAKDK
2263.3
2279.5
2263.6
+16(O)
+FIH
2260 2265 2270 2275 2280 2285
m/z

Fig. S6

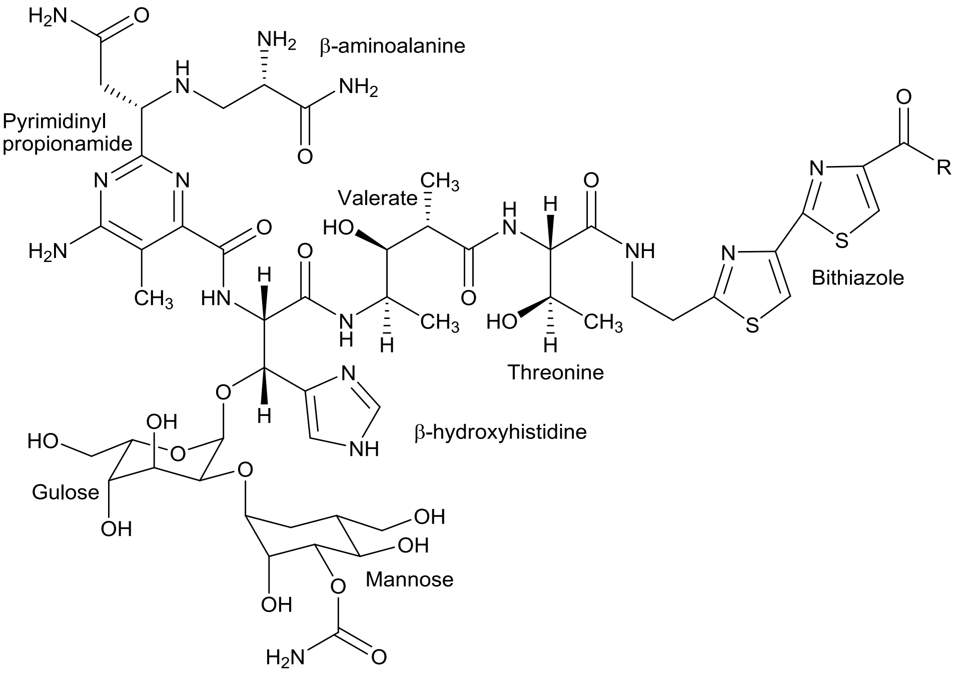
H2N
O
NH2
β-aminoalanine
H
N
NH2
Pyrimidinyl
propionamide
O
N
N
Valerate
CH3
O
HO
H
N
H
O
NH
N
S
R
Bithiazole
H2N
O
CH3
HN
H
O
N
H
H
CH3
O
HO
H
CH3
Threonine
N
S
O
H
N
NH
β-hydroxyhistidine
HO
OH
O
Gulose
OH
O
OH
OH
Mannose
OH
O
H2N
O

Fig. S7

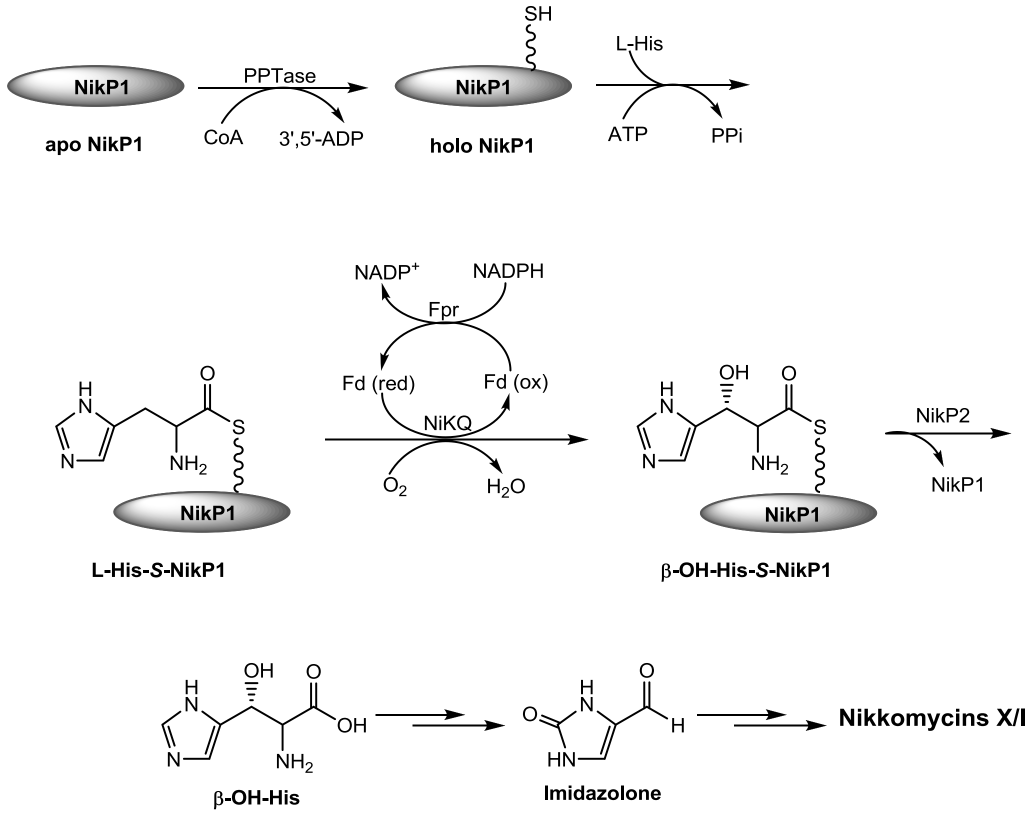
NikP1
apo NikP1
PPTase
CoA
3',5'-ADP
SH
holo NikP1
L-His
ATP
PPi
NADP+
NADPH
Fpr
Fd (red)
Fd (ox)
NiKQ
O2
H2O
L-His-S-NikP1
OH
β-OH-His-S-NikP1
NikP2
NikP1
β-OH-His
Imidazolone
Nikkomycins X/I
